# Evolutionary Profile of Mayaro Virus in the Americas: An Update into Genome Variability

**DOI:** 10.3390/v16050809

**Published:** 2024-05-20

**Authors:** Mikaela dos Santos Marinho, Giulia Magalhães Ferreira, Victória Riquena Grosche, Nilson Nicolau-Junior, Túlio de Lima Campos, Igor Andrade Santos, Ana Carolina Gomes Jardim

**Affiliations:** 1 Institute of Biomedical Sciences, ICBIM, Federal University of Uberlândia, Avenida Amazonas, 4C- Room 216, Umuarama, Uberlândia 38405-319, MG, Brazil; mikaelamarinho@ufu.br (M.d.S.M.); giumferreira95@gmail.com (G.M.F.); victoriagrosche@gmail.com (V.R.G.); 2 Institute of Biosciences, Humanities and Exact Sciences (Ibilce), São Paulo State University (Unesp), Campus São José do Rio Preto, São José do Rio Preto 15054-000, SP, Brazil; 3 Institute of Biotechnology, Federal University of Uberlândia, Uberlândia 38405-319, MG, Brazil; nicolaujr@ufu.br; 4 Aggeu Magalhães Institute (Fiocruz), Bioinformatics Core Facility, Recife 50740-465, PE, Brazil; tulio.campos@fiocruz.br

**Keywords:** arboviruses, genomic variability, phylogenetic analysis, Mayaro virus, Mayaro fever

## Abstract

The Mayaro virus (MAYV) is an arbovirus with emerging potential, though with a limited understanding of its epidemiology and evolution due to the lack of studies and surveillance. Here, we investigated 71 MAYV genome sequences from the Americas available at GenBank and characterized the phylogenetic relationship among virus strains. A phylogenetic analysis showed that sequences were grouped according to the genotypes L, D, and N. Genotype D sequences were closely related to sequences collected in adjacent years and from their respective countries, suggesting that isolates may have originated from circulating lineages. The coalescent analysis demonstrated similar results, indicating the continuous circulation of the virus between countries as well. An unidentified sequence from the USA was grouped with genotype D, suggesting the insertion of this genotype in the country. Furthermore, the recombination analysis detected homologous and three heterologous hybrids which presented an insertion into the nsP3 protein. Amino acid substitutions among sequences indicated selective pressure sites, suggesting viral adaptability. This also impacted the binding affinity between the E1–E2 protein complex and the Mxra8 receptor, associated with MAYV entry into human cells. These results provide information for a better understanding of genotypes circulating in the Americas.

## 1. Introduction

The Mayaro virus (MAYV) was first identified in 1954 in Trinidad and Tobago, in samples of febrile forest workers [1], later described as the etiologic agent of Mayaro fever. This disease is characterized by fever, headache, vomit, diarrhea, muscle pain, skin rashes, and long-lasting arthralgia [2]. In severe cases, MAYV infection can result in chronic polyarthritis, neurological complications, hemorrhage, and myocarditis, which can lead to death [3].

Mayaro fever in humans is mainly observed in Central and South Americas, predominantly in the Amazon Forest, Atlantic Forest, and nearby regions; however, it was also reported in subtropical regions [2]. Additionally, the disease presents dengue-like symptoms and it is present in endemic areas common to other arboviruses [4]; therefore, the differential diagnosis is difficult, which also contributes to the underreporting of Mayaro fever cases and genome sequences available in databanks [5]. Furthermore, there is no data about the circulating virus in other tropical regions, such as Africa and Asia [1].

The main vector for MAYV transmission is *Haemagogus* sp. mosquitoes [1]. However, the virus was also isolated from different nonurban arthropod genera such as the *Culex*, *Mansonia*, *Psorophora*, and *Sabethes*, as well as *Aedes albopictus* and *Aedes aegypti* species, demonstrating a high susceptibility to MAYV transmission, indicating an intense viral adaptability to new vectors [6]. Further, the distribution of these species in tropical and subtropical countries associated with globalization, climatic anomalies, deforestation, and the growing contact of humans with animal reservoirs/vectors is an aggravating factor, increasing the risk of MAYV dissemination [1,4]

**Figure 1:**
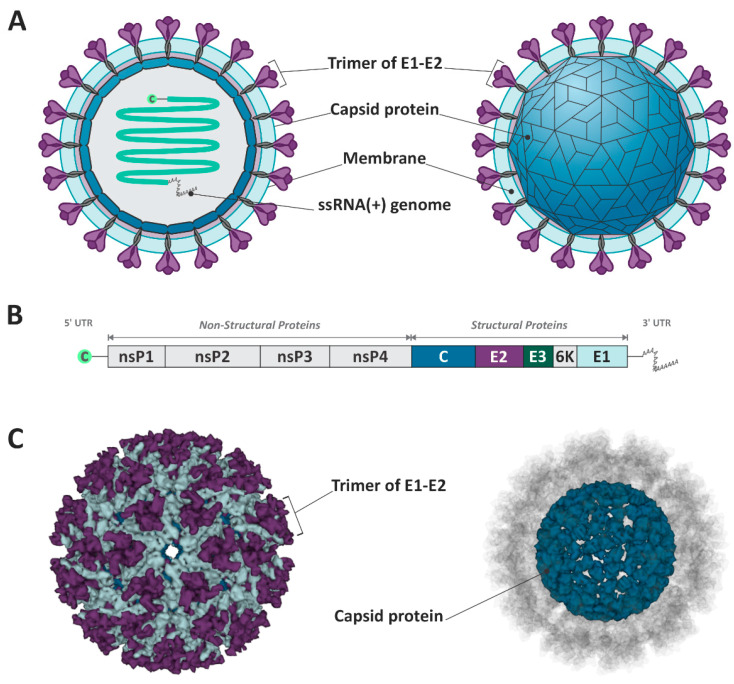
Schematic representation of Mayaro virus structure and genome. (**A**,**C**) The virion is composed of the E1–E2 trimmer, the capsid protein, and the ssRNA+. (**B**) The viral genome encodes structural and non-structural proteins. The figure design was based on Cryo-EM structure of mature MAYV. (PDB ID: 7KO8) [9].

The MAYV is a member of the *Togaviridae* family and *Alphavirus* genus and is classified into the genotypes D (widely dispersed), L (limited), and N (new), the latest being represented by a single sequence isolated in Peru [7]. The virion particle possesses a positive single-stranded RNA of approximately 11.5 kb, associated with a capsid protein (C), and involved in a host–lipid membrane envelope inserted of the E1 and E2 glycoproteins [8] (Figure 1). The MAYV genome is constituted of two open reading frames (ORFs), one in the 5′ UTR region, responsible for encoding the non-structural proteins (nsP1, nsP2, nsP3, and nsP4), and the second ORF after the nsP4 gene, which encodes the structural proteins (CP, E3, E2, TF, 6k, and E1) [3] (Figure 1).

It is also important to emphasize the higher rates of mutations within RNA viruses, which leads to an intra-host variability of the viral population [10]. Therefore, phylogenetic analyses represent important methodologies for developing epidemiological studies, allowing the correlation of data derived from different regions and presuming the history of the dissemination of viral variants [11]. Currently, only a few epidemiological studies on MAYV are reported, with a limited number of genome sequences available in databanks. Considering the severe symptoms caused by MAYV, and that this virus is responsible for a neglecting disease, allied with the possibility of the emergence of new variants, or additional transmitting vectors, the study of the viral genome variability represents an essential tool to investigate the virulence and transmissibility of the different viral genotypes [11].

In this context, genetic and phylogenetic analyses of MAYV complete-genome sequences were carried out here to investigate genomic variations and the phylogenetic relationships among the MAYV strains from samples collected in the Americas. Thus, an overview of the main features of the MAYV genomic variability was obtained, indicating evolutionary forces that could drive the evolution of the viral genome, and possibly contribute to viral adaptation.

## 2. Materials and Methods

### 2.1. Collection and Alignment of MAYV Genome Sequences

Sequences of the complete genome and complete CDS of MAYV (about 11,147 nucleotides) were collected from the GenBank database, and organized according to the year of collection, origin, genotype, host, length of the sequenced genetic material, article of reference, and reference of the genotype identification (Supplementary Table S1). The dataset containing all 71 sequences was submitted to alignment by the ClustalW multiple alignment method, using the program Bioedit 7.2 [12]. The NCBI sequence NC_003417.1 was used as a reference sequence, and the parameters of alignment used were defined by the program’s default configuration.

### 2.2. Detection of Individual Recombination Events

Due to the identification of MAYV sequences initially classified as genotype L as L/D hybrids by previous studies, KY985361.1 (Haiti, 2014) [13] KX496990.1 (Haiti, 2015), and KT818520.1 (Brazil, 2014) [14], the 71 genome sequences of MAYV were evaluated for potential recombination events using Recombination Detection Program 4 (RDP4) [15]. RDP4 was used with the default parameters, applying all available methods through the full exploratory recombination scan (Recombination Detection Program, Bootscan, MaxChi, GENECONV, Chimaera, SiScan, 3Seq, LARD, and Phylpro). The recombination breakpoints suggested by RPD4 were used to identify the potential recombinant sequences.

### 2.3. Phylogenetic Analysis of MAYV Genomic Sequences

To this, the recombinant regions of MAYV strains were removed and the phylogenetic tree was reconstructed employing PhyML 3.0 through the ATGC- Montpellier Bioinformatics Platform [16]. The method employed was maximum-likelihood analysis (ML), and the GTR + G model of substitution, as determined by the Smart Model Selection in PhyML [17]. A thousand replicates were used to test the support given by the data to the clusters of the tree topology, and bootstrap values > 70 were considered significant (McCormack & Clewley, 2002). The phylogenetic tree was edited using the program FigTree v.1 4.4 [12].

### 2.4. Coalescent Analysis

The optimal substitution model for the alignment was assessed using ModelFinder in IQ-TREE v2.1.4-beta (GTR + F + I + G4), generating a maximum likelihood tree. Root-to-tip correlation (>0.8) for the consensus tree was inspected in TempEst v1.5.3 with best-fitting root and residual mean square function, suggesting a strict molecular clock. The alignment was then imported into BEAUti v1.10.4 using tip dates parsed (year only) from the sequence names. The GTR substitution model was selected with empirical base frequencies and gamma (4 categories) plus invariant sites. We selected a strict clock type and a coalescent tree, previously, with constant size, and a random starting tree. Other parameters remained unchanged. Further, a Markov chain Monte Carlo with 100 million interactions using BEAST v1.10.4 was performed, logging the results at every 1000 interactions. The convergence of the model parameters was inspected using Tracer v1.7.2, confirming that the ESS for each statistic was >400 at the end of the runs. Tree annotator v1.10.4 was then employed to generate a maximum clade credibility tree with median heights for all trees generated, following the removal of the first 10% as burn-in. The final tree was inspected, and a figure was generated using IcyTree [18].

### 2.5. Analysis of Amino Acid Substitutions and Selection Pressure

Sequences were aligned according to their genotype, resulting in two datasets: (1) sequences identified as genotype D, using DQ001069.1 as the reference sequence, and (2) sequences identified as genotypes L and L/D, employing as reference the genotype L sequence NC_003417.1. The alignments were edited according to the protein-coding regions and the translation was performed. Amino acid substitutions were analyzed in comparison to the reference sequence. The selective pressure analysis was performed on MAYV protein-coding sequence subsets through the Datamonkey Adaptive Evolution Server [19], to identify and localize statistically supported positive and negative selective pressure sites, as previously described [20]. To predict the impact of each substitution in protein selection, the fast unconstrained Bayesian approximation (FUBAR) model was used, which applies a maximum-likelihood (ML) approach to infer nonsynonymous (dN) and synonymous (dS) substitution rates on a per-site basis for a given coding alignment and corresponding phylogeny [21]. In addition, the fixed effects likelihood (FEL) model was employed to compare the results obtained. The selection pressure for each site was considered constant throughout the entire phylogeny. The statistically supported selective pressure sites considering Bayes factor (BF) > 30 and *p* value < 0.05 were reported.

### 2.6. Protein Modeling and Docking

The structure of the E1 and E2 from MAYV from different isolates were modeled together with its human natural ligand Mxra8 using SWISS-MODEL server [22]. In order to construct the complexes, a template from an electron cryo-microscopy of Chikungunya virus spike protein in complex with mouse Mxra8 receptor (PDB id:6NK7) was used. After the complexes’ generation, the results and the template structure (6NK7) were submitted to two servers, PRODIGY [23] for binding affinity prediction and PLIP [24] for hydrophobic, hydrogen bond, and salt bridge interactions. The best E1E2-Mxra8 binding affinity complex was presented in a 3D view using ChimeraX [25].

## 3. Results

### 3.1. Epidemiological Features of MAYV in the Americas

**Figure 2:**
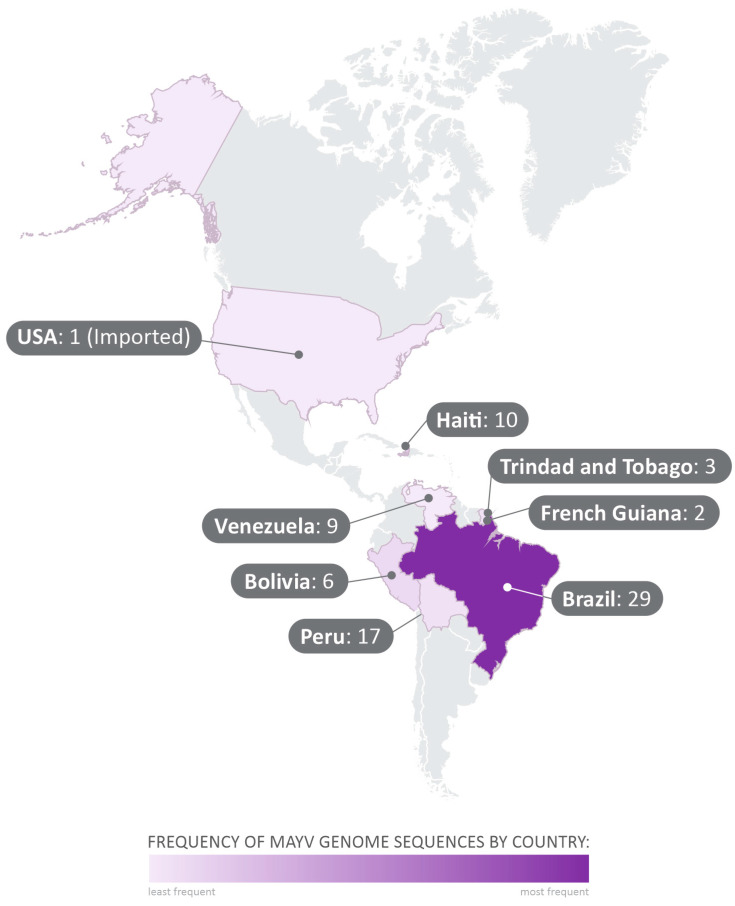
Geographical distribution of the analyzed sequences collected from GenBank. The 71 complete-genome sequences selected in the study were collected between 1954 and 2015, in Brazil, Trinidad and Tobago, Haiti, United States of America (USA), Bolivia, Peru, French Guiana, and Venezuela.

The 71 complete genome sequences obtained from GenBank were collected between 1954 and 2016, in Brazil, Trinidad and Tobago, Haiti, United States of America (USA), Bolivia, Peru, French Guiana, and Venezuela (Figure 2). All MAYV sequences and the related data employed in this study are shown in Supplementary Table S1.

### 3.2. Individual Recombination Events Detected among MAYV Sequences

**Figure 3:**
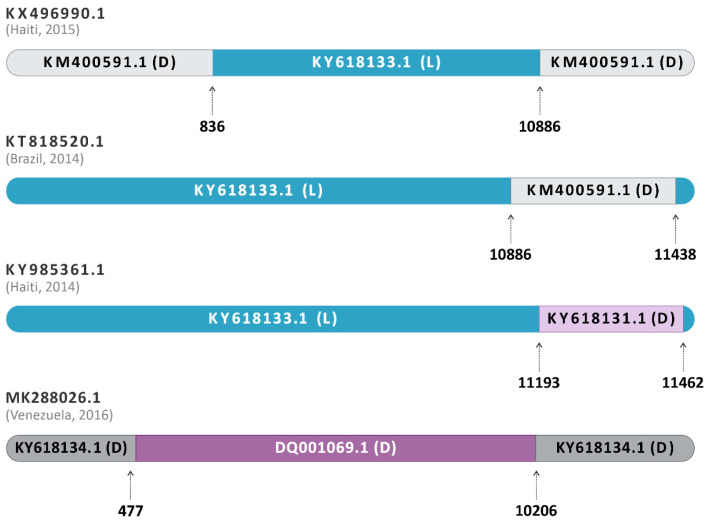
Schematic representation of the recombination event in sequences classified as hybrids. The dataset containing the 71 sequences was submitted to the RDP4 software program v.4.101. KY985361.1, from Haiti, 2014, was identified as an L/D hybrid between the major parent KY618133.1 of Genotype L, Brazil, 1988 (blue) and the minor parent KY618131.1 of Genotype D, Brazil, 1978 (purple). The other hybrids, KX496990.1 (Haiti, 2015) and KT818520.1 (Brazil, 2014), have their L major parents represented in blue and the D minor parents represented in grey. The homologous hybrid MK288026.1 (Venezuela, 2016) had DQ001069.1 (genotype D, French Guiana, 1998) as a major parent and KY618134.1 (genotype D, Brazil, 1991) as a minor parent.

The submission of the complete alignment with all analyzed sequences from genotypes D, L, and L/D on RDP4 showed evidence of a heterologous recombination among the sequences KY985361.1 (Haiti, 2014), KX496990.1 (Haiti, 2015), and KT818520.1 (Brazil, 2014) (Figure 3). The recombination event was positive in all seven methods listed above (*p* < 0.05), being inconclusive only for LARD and Phylpro, also used in the analysis of the sequences (Supplementary Table S2).

Furthermore, the sequence MK288026.1 (Venezuela, 2016) was identified as a homologous hybrid between DQ001069.1 (genotype D, French Guiana, 1998) and KY618134.1 (genotype D, Brazil, 1991) (Figure 3). The isolate DQ001069.1 was the most closely related to the likely hybrid, being a major parent, and KY618134.1 was identified as the minor parent. In addition, the sequence KT818520.1 (Brasil, 2014) was identified as an L/D hybrid, with supported evidence of a similar origin or same recombination event as KX496990.1 (Haiti, 2015) (Figure 3), as previously described by Mavian and collaborators [14]. Additionally, KY985361.1 (Haiti, 2014), also previously reported as genotype L [2], and subsequently identified as a hybrid [13], was also identified here as an L/D hybrid by all RDP4 conclusive analyses. The sequence was identified as originating from the major parent KY618133.1 (Genotype L, Brazil, 1988) and the minor parent KY618131.1 (Genotype D, Brazil, 1978) (Figure 3). Interestingly, as with the other sequences, KY985361.1 (Haiti, 2014) also showed a higher similarity to its major parent of genotype L. All the recombination events mentioned presented *p*-values ≤ 4523 × 10^−2^ (Supplementary Table S2), being considered potential recombinant events.

### 3.3. Phylogenetic Relationships and Coalescent Analysis of MAYV Complete Genomic Sequences

**Figure 4:**
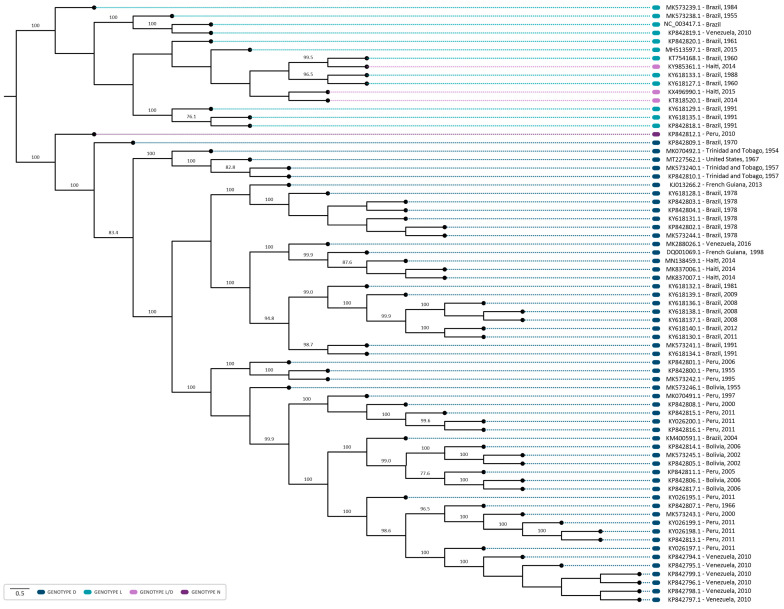
Midpoint-rooted phylogenetic tree reconstruction using 71 sequences of the MAYV complete genome. The sequences were aligned employing the ClustalW multiple alignment method and the phylogenetic tree was reconstructed employing PhyML 3.0, through the ATGC- Montpellier Bioinformatics Platform, using maximum-likelihood analysis (ML), and the GTR + G + I model of substitution, as determined by the Smart Model Selection in PhyML. The NCBI reference sequence NC_003417 (Genotype L) was used as the reference sequence. The sequences from the genotypes D, L, L/D, and N are highlighted in blue, light blue, lilac, and purple, respectively. A thousand replicates were used to test the support given by the data to the clusters of the tree topology, and bootstrap values > 70 were considered significant.

**Figure 5:**
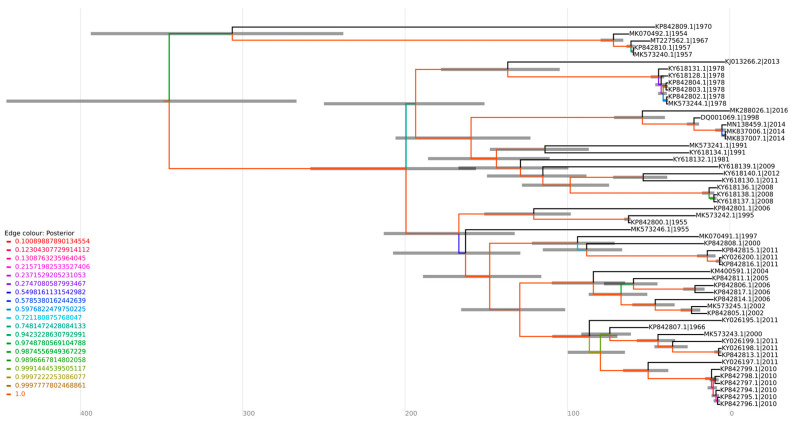
Coalescent analysis of genotype D sequences. The sequences were aligned employing the ClustalW multiple alignment method, and the optimal substitution model for the alignment was assessed using ModelFinder in IQ-TREE v2.1.4-beta (GTR + F + I + G4), generating a maximum likelihood tree. The sequences were submitted to a strict molecular clock using the GTR substitution model.

The phylogenetic analysis revealed that sequences were grouped according to the MAYV genotypes (D, L, and N) (Figure 4). Twelve sequences were identified as genotype L, fifty-five as genotype D, and, as previously reported, one was identified as genotype N and three as hybrids L/D [13,14]. Furthermore, in both coalescent and phylogenetic trees, the characterized sequences exhibited a closer grouping with those isolated in the same country and in proximate years (Figure 4 and Figure 5). It suggests that the clustered sequences may have originated from circulating lineages due to the phylogenetic and genomic similarity among the isolates. In this sense, the biggest clade comprehending sequences isolated from Brazil (1981–2011) also present phylogenetic similarity (bootstrap of 94.8) and share the same ancestry, with a time to the most recent common ancestor (tMRCA of 143.75 and posterior probability of 1), indicating the persistent circulation of the virus. Intriguingly, certain groupings within the clusters were associated with notable outbreaks, such as those in Venezuela (2010) and Peru (2010–2013) [26]. It is noteworthy that these strains share a common ancestry with a tMRCA of 73.713 and a high posterior probability of 99.97%. Similarly, six sequences isolated in Brazil (1978) and one from French Guiana (2013) were grouped in the same clade (bootstrap of 100%) and had the same ancestor with a tMRCA of 136.76 and posterior probability of 1, which could indicate the continuous circulation of the virus between countries (Figure 4 and Figure 5).

Furthermore, the sequence MT227562.1, isolated from a sample of an allochthonous case in Louisiana (USA), which had no previous description regarding its genotype, formed a cluster with sequences of the genotype D (Figure 4), suggesting this sequence as belonging to this genotype. This strain was closely related to isolates MK070492.1 (1954), MK573240.1 (1957), and MK5732402.1 (1957) from Trinidad and Tobago in the phylogenetic tree, supported by a bootstrap of 100%. The same outcome was observed in a coalescent analysis, with a tMRCA of 71.663 and a posterior probability of 1, indicating that the sequences share the same origin (Figure 5).

Regarding the analysis of genotypes L and N, the lack of sequencing data hindered the undertaking of a coalescent analysis for these specific sequences. The topology of the phylogenetic tree demonstrated that L/D sequences formed a cluster with sequences of the L genotype, suggesting a higher genomic similarity. The sequence KX496990.1–Haiti, 2015 (L/D) showed a phylogenetic proximity with one of the L/D sequences from Brazil (KT818520.1, 2014), (Figure 4). Interestingly, these sequences were collected in subsequent years (2014–2015), which may suggest a possible route of transmission of MAYV.

### 3.4. Follow-Up of the Amino Acid Substitutions and Selection Pressure among MAYV Sequences

**Figure 6:**
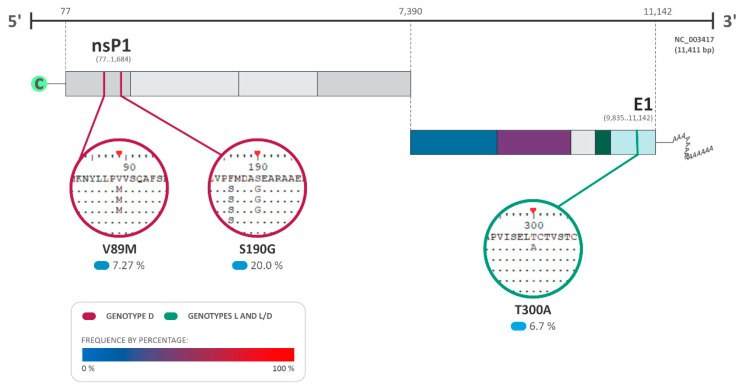
Amino acid substitutions and selection pressure among MAYV sequences. The most relevant amino acid substitutions among the sequences of the complete genome of MAYV that presented positive selection pressure with a *p*-value under 0.05 and a Bayes factor (BF) > 30 are shown (BF of 30–100: very strong evidence for positive selection pressure). DQ001069.1 isolated from a sample of *Homo sapiens* in French Guiana was used as the reference sequence for genotype D. The NCBI reference sequence NC_003417.1 isolated from Brazil was used for genotype L and L/D. The frequency of the substitutions is presented as a percentage, where 0% is represented in light blue and 100% in red.

The analysis of the amino acid substitutions and their effects on viral protein structures was performed using the fixed effects likelihood (FEL) and fast unconstrained Bayesian approximation (FUBAR) DataMonkey softwares v. 2.0 to detect the presence of neutral (synonymous), adaptative (non-synonymous), and purifying (non-synonymous) substitutions, under a *p*-value < 0.05 (Table 1; Figure 6). Additionally, the Bayes factor (BF) was also employed to measure the relative evidence of substitutions, with values higher than 30 characterized as very strong evidence of the positive selection pressure hypothesis at the site [27]. As a result, several substitutions were identified among the MAYV sequences with three of those sites identified to be under positive/adaptative selection (two in genotype D and one in genotype L–L/D) (Table 1; Figure 6). For genotype D, the amino acid substitutions V89M and S190G were observed in nsP1, with a Bayes factor (BF) of 10.065 and 489.759, respectively (Table 1; Figure 6). On the other hand, genotype L demonstrated amino acid substitution in the E1 protein (T300A: BF of 36.041) (Table 1; Figure 3).

The sequences of the MAYV genome also presented amino acid substitutions characterized as neutral or under negative selection pressure in all viral proteins. The prevalence of amino acid substitutions was high across most MAYV proteins, particularly within sequences belonging to genotype D, which outnumbered those of genotype L and L/D with 55 sequences compared to 12 sequences, respectively. 

**Figure 7:**
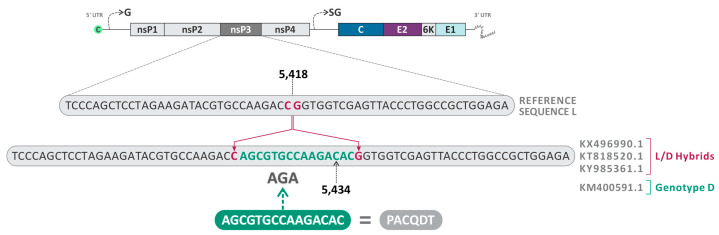
Schematic representation of the nucleotide insertion in the nsP3 observed in the sequences KY985361.1 (Haiti, 2014), KX496990.1 (Haiti, 2015), KT818520.1 (Brazil, 2014), and KM400591.1 (Brazil, 2014).

The insertion of the amino acids ACQDT (nucleotide sequence: AGCGTGCCAAGACAC) between the amino acid positions 443–444 of the nsP3 was found in three sequences already described as L/D (KX496990.1—Haiti, 2015; KT818520.1—Brazil, 2014; and KY985361.1—Haiti, 2014) (Figure 7). Interestingly, the isolate KM400591.1 (Brazil, 2014), classified as MAYV genotype D, also presented the same insertion and was identified as a minor parent for the heterologous hybrid KX496990.1 (Haiti, 2015), which also presented this characteristic. 

### 3.5. Molecular Modeling and Docking

**Figure 8:**
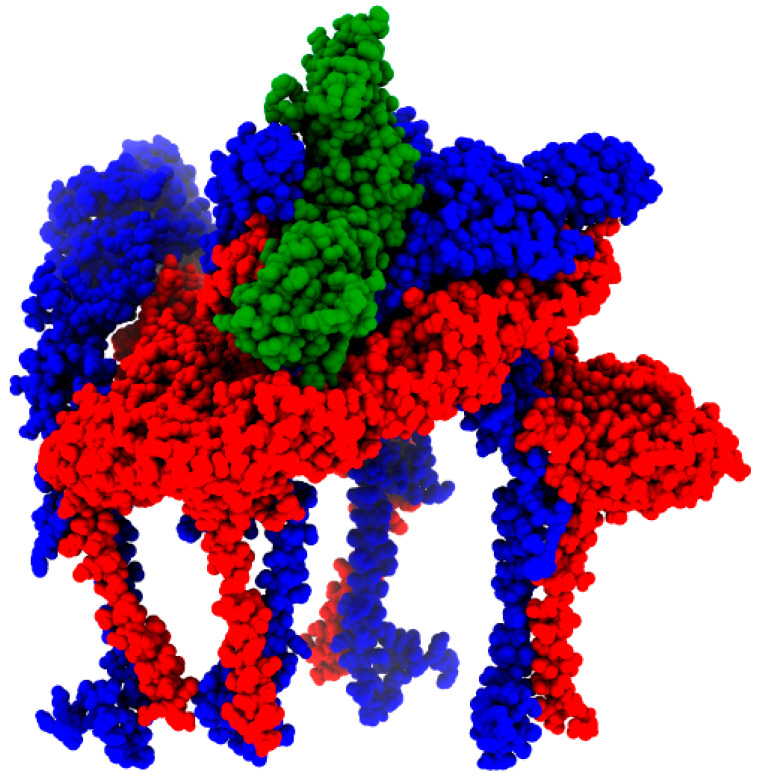
MAYV E1 (red) and E2 (blue) octamer complexed with human Mxra8 (green). The result was generated by MAYV isolate KT818520.1 modeling.

Binding affinity predictions showed that the isolate KT818520.1 (Figure 8) presented the best binding affinity (lowest energy) result among the complexes; however, the reference sequence NC_003417.1 presented the highest number of interactions overall (Table 2). Interestingly, the sequence MK573239.1, featuring a positive selection site (T300A) on the E1 protein, displayed a binding energy slightly lower than the reference sequence. Another finding was the lower binding affinity observed in the MAYV heterologous hybrids KT818520.1 and KX496990.1, originating from the same parental strains. This affinity was notably lower than that of KY985361.1, which stemmed from the same parental strain of genotype L but was a distinct strain from genotype D. Additionally, the complex of Chikungunya virus E1–E2 and mouse Mxra8 obtained from the protein data bank, used as template in the modeling phase, showed the worst binding affinity (highest energy) and a small number of salt bridges when compared to the human complexes (Table 2).

## 4. Discussion

The incidence of MAYV infections has been mainly described in Latin America, with most Mayaro fever cases reported in Brazil [6]. In agreement with these data, the majority of genomic sequences analyzed here were obtained from samples collected in Brazil, which included MAYV isolates of the genotypes L, D, and L/D. Although most cases of Mayaro fever have been observed in South America, there are reports of outbreaks in Haiti, indicating that the virus is also circulating in Central America [28]. Mayaro fever was likewise reported in Europe [3]; however, there are no epidemiological studies that describe the introduction of the genotypes in the region, or whether the infection has spread by local vectors. Furthermore, to the best of our knowledge, there is no current data on the circulation of MAYV in Africa and Asia, due to the lack of data reported on these continents [1]. It is also observed that there is poorly described information concerning the MAYV strains, such as the N genotype, isolated in Peru in 2010, with no current description of its dissemination degree [2]. Therefore, studies that aim to investigate the epidemiological characteristics of MAYV worldwide, as well as the genetic and phylogenetic relationship among circulating viral strains, are essential.

Here, we analyzed the MAYV genomic variations in sequences from different regions of America and characterized the phylogenetic relationship among these virus strains, to investigate the main evolutionary forces driving the evolution of the viral genome and their possible effect on viral activity, associating them with the dissemination of MAYV. Concerning the genetic variability among the viral genome sequences, it is important to emphasize the higher genetic plasticity and mutational rate of RNA viruses, which leads to an intra-host variability of the viral population, as well as the emergence of new species, genera, and/or new viral families [10,29]. Moreover, arboviruses exhibit bottleneck-mediated drift due to constriction points during vector infection and transmission, as well as within the transmission cycle itself [30]. This dynamic can significantly impact the future of arboviral epidemics and disease outcomes. Herein, a high incidence of polymorphisms was found in MAYV sequences, with several of those substitutions classified within purifying selection sites, which demonstrates mechanisms to eliminate deleterious mutations and maintain the accumulated characteristics of the virus [31]. These polymorphisms were widely observed when the genomic regions related to most viral proteins were evaluated, some of them directly involved in viral replication. This demonstrates an attempt to constrain the variability at the protein level since the negative selection tends to conserve the sequence and the structural characteristics of active motifs of the proteins [32]. The proteins from the replicative complex as well as the structural proteins were found under negative pressure in several positions in almost all analyzed sequences, which may suggest that the circulating genotypes have an adapted fitness. The genetic variability and the evolution potential within a population are the product of a balance of selection and genetic drift [32], and, therefore, further analysis regarding the up and down fluctuation of viral fitness is necessary to achieve knowledge of the gain or loss of function due to substitutions observed over the viral genome. However, the restricted number of sequences of MAYV poses a delay in the analyses of the persistence and disappearance of polymorphisms over time, as well as the difficulty in the suggestion of adaptability to the host, higher transmissibility, or immune escape [33].

Alternatively, we also identified positive selection pressure sites, which drives the accumulation of mutations or genetic variations that confer advantages in the viral population, such as immune evasion and host-jumps [34]. Among the sequences of the genotype L, it was observed only in the sequence MK573239.1 (Brazil, 1984) at the E1 protein. Interestingly, the substitution into the E1 (T300L) protein was not identified by FEL, which utilizes a maximum-likelihood (ML) method to spot genetic variations [21]. In contrast, the polymorphism was identified through the analysis carried out with FUBAR, which produces similar information to FEL but employs a Bayesian approach to infer nonsynonymous (dN) and synonymous (dS) substitution rates [21]. Interestingly, a single mutation in the CHIKV E1 protein (A226V) allowed the virus to infect a new vector species, *Aedes albopictus* [35,36], facilitating the spread of the virus and leading to outbreaks in non-endemic areas [35]. In this context, given the susceptibility of *Aedes albopictus* and other mosquito species to MAYV infection, coupled with this new mutation found in MAYV E1 protein, it is possible to suggest that a similar outcome might be expected. In addition, the molecular modeling indicated that this positive selection site induced a slightly lower binding energy between the E1–E2 trimmer and the receptor Mxra8, resulting in a higher affinity. However, this polymorphism was not found in other isolates, which may suggest that these substitutions did not sustain their circulation at low frequency. In this sense, these mutations can also be associated with immune response escape, as demonstrated by Earnest and colleagues [37]. Their study evaluated different monoclonal IgG antibodies against the MAYV strain containing substitutions on the E1 protein and only 4 of 18 antibodies demonstrated elite neutralizing activity against this virus [37]. Similarly, Rangel and colleagues pointed out that the single mutations V156A and K211T can influence antibody neutralization against CHIKV and increase virus replication and virulence in mice, emphasizing the role of the mutation in escaping immune response and infectivity increase [36].

In addition, amino acid substitutions identified in the genomic regions of the nsP1 of sequences of genotype D were found to be under positive selection pressure (Figure 2, Supplementary Table S2), suggesting the possible viral adaptability and improvement of fitness during viral replication. The nsP1 protein is directly involved in the initiation of virus replication, responsible for interacting with the inner face of the plasma membrane and promoting the formation of the spherules, which are vesicles where the replicative process initiates [38]. Even though the presence of polymorphisms by positive selective pressure is relevant due to its impact on virus evolution, the low level of positive selection suggests that the amino acid changes have been a result of random genetic drift. Interestingly, the same pattern was seen in other RNA viruses such as the West Nile virus and Hepatitis C virus [39].

Another finding was the insertion of the amino acids ACQDT into nsP3 found in three sequences identified as L/D hybrids (KX496990.1—Haiti, 2015; KT818520.1—Brazil, 2014; and KY985361.1—Haiti, 2014) and one genotype D strain (KM400591.1—Brazil, 2014). A study conducted by Neyret and colleagues demonstrated that the deletion of an upstream FGAP sequence in the G3BP binding sequence present in nsP3 damages the nsP3/G3BP interaction and infectivity of MAYV, resulting in lower rates of viral replication [40]. Differently, in our study, we observed insertions in nsP3 located between positions 443–444, close to the canonical binding site of nsP3 G3BP (associated with residues between 456–478). In this sense, considering that nsP3 plays a critical role in the infectivity of alphaviruses, this insertion may favor the replication of the L/D strains or make them unfeasible.

Our phylogenetic analysis demonstrated that the sequence MT227562.1 was classified as genotype D. This sequence was obtained from a sample of an allochthonous case in Louisiana (USA), isolated from *Icterus spurius*, a bird with a migratory route between Central America, Colombia/Venezuela, and the USA [41]. Furthermore, within the phylogenetic tree, this sequence exhibits a close grouping with sequences isolated in Trinidad and Tobago between 1954 and 1957. Remarkably, our coalescent analysis indicated that these sequences also present a common ancestral source. Therefore, it is possible to infer that the bird was infected in nearby regions of Trinidad and Tobago, and, during its migration to the USA, this led to the insertion of the genotype in the region, given the predominance of genotype D in the Americas.

Furthermore, the L/D sequences were phylogenetically closer to the sequences of genotype L, demonstrating a higher genomic similarity with the L genotype sequences, as indicated by previous studies [13,14]. It is important to emphasize that L/D hybrids were initially classified as members of the L genotype, prior to the recognition of the L/D recombination [42,43]. Among these, KT818520.1 (Brazil, 2014) and KX496990.1 (Haiti, 2015) were identified by RDP4 as potentially originating from the same recombination event, which may suggest a possible route of dissemination of MAYV. Furthermore, the emergence of MAYV recombinants/hybrids demonstrates the potential for the emergence of a future new genotype and its dissemination, highlighting the need for genomic surveillance for this pathogen. It has also been described for other RNA viruses, such as in Chikungunya virus (CHIKV), that homologous recombination resulted in cross-species transmission, and, therefore, impacted virus evolution, emergence, and epidemiology [44]. In addition, the interspecies recombination was also identified in other types of Alphavirus, such as Eastern equine encephalitis virus (EEEV) and Sindbis-like virus (SIN), originating from the Western equine encephalitis virus (WEEV), Highlands J virus (HJV), and Fort Morgan virus (FMV) [45]. In this context, MAYV recombinants could emerge and spread as new strains with an increased fitness in human and *Haemagogus* vector hosts, as well as adapt to new hosts.

## 5. Conclusions

In conclusion, the data presented here demonstrate that sequences of the complete genome of MAYV are under negative and positive selection pressures, emphasized by the high number of purifying selection sites, that might result in the emergence of new strains. Moreover, the study highlights the concerning absence of surveillance efforts for MAYV. Despite sequences being collected across decades, they share common ancestors and exhibit phylogenetic proximity, suggesting the sustained circulation of the virus. In this sense, it is essential to diagnose and sequence the MAYV genome to characterize the virus adaptation and transmissibility. Therefore, these data can be used to define the degree of MAYV dissemination and implement protective measures to manage Mayaro fever.

## Figures and Tables

**Table 1:** Sites of the MAYV genome under positive selection pressure based on FUBAR and FEL that presented amino acid substitutions.

Genotype	Protein	Site	Method	Alpha	Beta	Bayes Factor	*p*-Value
**D**	nsp1	89	FUBAR	0.992	11.058	10.065	<0.05
			FEL	-	-	-	-
		190	FUBAR	0.619	15.575	489.759	<0.05
			FEL	0.000	8.352	n/a	0.0144
**L**	E1	300	FUBAR	2.252	28.971	36.041	<0.05
			FEL	-	-	-	-

**Table 2:** E1E2-Mxra8 binding affinity and number of interactions from the different complexes.

Genbank ID	Binding Affinity ΔG (kcal mol^−1^)	Hydrophobic Interaction	Hydrogen Bond	Salt Bridge
KT818520.1	−13.2	8	11	6
KX496990.1	−13.1	9	10	7
MT227562.1	−13.1	9	11	7
MK573239.1	−13.1	9	11	7
NC_003417.1	−13.0	9	12	7
KY985361.1	12.9	9	12	6
DQ001069.1	−12.9	8	10	7
PDB: 6NK7	−11.2	9	12	1

## Data Availability

All the sequencing data used in this article can be found at PubMed Genbank database.

## Supplementary Materials

The following supporting information can be downloaded at: https://www.mdpi.com/article/10.3390/v16050809/s1, Table S1: MAYV sequence data from the analyzed sequences. Contains year of collection, origin, genotype, host, size of the sequenced genetic material, reference article for sequencing, and reference of genotype identification.; Table S2: Recombination confirmation provided by RDP4. Presents the recombination event, methods applied, and *p*-value.
